# Supplementary material for: Effectiveness of an Active and Continuous Surveillance Program for Intensive Care Units Infections Based on the EPIC III (Extended Prevalence of Infection in Intensive Care) Approach
Source: J Clin Med. 2022 Apr 28;11(9):2482. doi: 10.3390/jcm11092482 (PMC9101920; doi:10.3390/jcm11092482)
Supplement: Supplementary file 1 [file jcm-11-02482-s001.zip › CFR_8.4.22.pdf]

STUDIO SPRINT - CFR

STUDY DAY -- __/__/____								
ICU	□ general	□ cardiosurg	□ traumatologic	□ post-op	□ neurosurg	□ pediatric	□ emergency dept.	□ gyn/obst

Patient				
	ID num	hospital admission __/__/____	ICU admission __/__/____	
	Initials	Age	□ male □ female	Weight	Height

Admission Type	□ medical	□ surgical election	□ surgical emergency	□ trauma		
Origin	□ other hospital	□ DEA	□ medical dept.	□ surgical dept.	□ other	
Reason for Adm	□ respiratory	□ cardiovascular	□ neurological	□ trauma	□ surveillance	□ other
Main Diagnosis						
Other Diagnosis						
Comorbidities	□ active solid cancer	□ inactive solid cancer (<5y)	□ inactive solid cancer (>5y)	□ metastatic cancer	□ lymphoma	□ other heamatological cancer
	□ immunosuppressive therapy (<3months)	□ immunosuppressive therapy (>3months)	□ chronic kidney failure	□ COPD	□ heart failure (NYHA 3-4)	□ previous cardiac disease
	□ solid organ transplantation	□ neurodegenerative disease	□ IDDM	□ NIDDM	□ chronic dialysis	□ cirrhosis (CHILD B-C)
	□ CT/RT (<6months)	□ CT/RT (>6months)	□ allogenic stem cells transplant	□ autologous stem cells transplant	□ HIV infection	

**Temp (°C)**	min	max	**HR (bpm)**	min	max
**SBP (mmHg)**	min	max	**MAP (mmHg)**	min	max

**Vasopressors/Inotropes** □ yes □ no	1.	Dose (mcg/kg/min)
	2.	Dose (mcg/kg/min)
	3.	Dose (mcg/kg/min)
	4.	Dose (mcg/kg/min)

**Respiratory**	□ spontaneous breathing	□ mechanical ventilation		**RR (/min)**	min	max		
**pH**	min	max	**PaO_2** (mmHg)	min	max	**$PaCO_2$** (mmHg)	min	max
**HCO_3** (mmol/l)	min	max	**FiO_2** (%)	min	max	**PaO_2/FiO_2**	min	max
**WBC** ($10^3/mm^3$)	min	max	**Hb** (g/dl)	min	max	**PLTS** ($10^3/mm^3$)	min	max
**Hct** (%)	min	max	**Crea** (mg/dl)	min	max	**Lac** (mmol/l)	min	max
**Na^+** (mmol/l)	min	max	**K^+** (mmol/l)	min	max			
**Bilirubine** (mg/dl)	max		**Urea** (mg/dl)	max		**Urinary Output** (ml/24h)		

**GCS**	during sedation	EVM	E	V	M	**SOFA**	
	neurologic window	EVM	E	V	M	**SOFAq**	

**Invasive ventilation**	□ yes □ no	start date __/__/____	**Tracheostomy**	□ yes □ no	date __/__/____
**CPAP/NIV**	□ yes □ no	start date __/__/____	**Coaxial catheter**	□ yes □ no	date __/__/____
**HF nasal O_2**	□ yes □ no	start date __/__/____	**Urinary catheter**	□ yes □ no	date __/__/____
**ECMO**	□ yes □ no	start date __/__/____	**DVE**	□ yes □ no	date __/__/____
**CVC_1**	□ yes □ no	date __/__/____	lumens	site	
**CVC_2**	□ yes □ no	date __/__/____	lumens	site	
**Intermittent hemodialysis**	□ yes □ no		**CRRT**	□ yes □ no	
**Septic Shock**	□ yes □ no		**Hyperlactacidemia**	□ yes □ no	

INFECTION SECTION		
**ATB prophylaxis** □ yes □ no	1.	
	2.	
	3.	
□ GE decontamination	□ oral Chlorhexidine	□ nasal Mupirocin

**INFECTION 1** □ yes □ no					
**Site**	□ respiratory system	□ abdomen	□ circulation	□ kidneys	□ skin
	□ CNS	□ catheter-related	□ genito-urinary	□ other	
**Evidence**[1]	□ certain	□ probable	□ feasible		
**Acquisition mode**[2]	□ community-acquired	□ hospital-acquired/health care-associated	□ ICU-acquired		
**Positive isolate** □ yes □ no	bug 1				
	bug 2				
	bug 3				

**INFECTION 2** □ yes □ no					
**Site**	□ respiratory system	□ abdomen	□ circulation	□ kidneys	□ skin
	□ CNS	□ catheter-related	□ genito-urinary	□ other	
**Evidence**[1]	□ certain	□ probable	□ feasible		
**Acquisition mode**[2]	□ community-acquired	□ hospital-acquired/health care-associated	□ ICU-acquired		
**Positive isolate** □ yes □ no	bug 1				
	bug 2				
	bug 3				

**INFECTION 3** □ yes □ no					
**Site**	□ respiratory system	□ abdomen	□ circulation	□ kidneys	□ skin
	□ CNS	□ catheter-related	□ genito-urinary	□ other	
**Evidence**[1]	□ certain	□ probable	□ feasible		
**Acquisition mode**[2]	□ community-acquired	□ hospital-acquired/health care-associated	□ ICU-acquired		
**Positive isolate** □ yes □ no	bug 1				
	bug 2				
	bug 3				

**INFECTION 4** □ yes □ no					
**Site**	□ respiratory system	□ abdomen	□ circulation	□ kidneys	□ skin
	□ CNS	□ catheter-related	□ genito-urinary	□ other	
**Evidence**[1]	□ certain	□ probable	□ feasible		
**Acquisition mode**[2]	□ community-acquired	□ hospital-acquired/health care-associated	□ ICU-acquired		
**Positive isolate** □ yes □ no	bug 1				
	bug 2				
	bug 3				

**ATB therapy** □ yes □ no	1.
	2.
	3.
	4.
	5.
**ATB therapy for recent infection** □ yes □ no	

FOLLOW UP						
**Dismissal**	ICU dismissal __/__/____			hospital dismissal __/__/____		
	ICU Dismissal to	□ other ICU	□ intermediate unit	□ department	□ dead	□ other
**Hospital dismissal before 60 days**	□ yes	□ no				
**Outcome at 60 days**	□ alive	□ dead				

BUG legenda

100	gram positivi
101	staphylococcus aureus, sensibilità/resistenza non nota
102	staphylococcus aureus sensibile alla meticillina (MSSA)
103	staphylococcus aureus resistente alla meticillina (MRSA)
104	staphylococcus aureus resistente a linezolid
105	staphylococcus aureus con resistenza totale o intermedia alla vancomicina (VISA)
106	stafilococchi coagulasi-negativi (epidermidis, emolitico,…) sensibilità/resistenza non nota
107	stafilococchi coagulasi-negativi (epidermidis, emolitico,…) sensibili alla meticillina
108	stafilococchi coagulasi-negativi (epidermidis, emolitico,…) resistenti alla meticillina
109	streptococcus gruppo D (enterococcus faecalis, faecium) sensibilità/resistenza non nota
110	streptococcus gruppo D (enterococcus faecalis, faecium) sensibili alla vancomicina
111	streptococcus gruppo D (enterococcus faecalis, faecium) resistenza totale o intermedia alla vancomicina (VRE)
112	strepotococcus A, B, C, gruppo G
113	streptococcus peumoniae, sensibilità/resistenza non nota
114	streptococcus pneumoniae resistente ai macrolidi
115	streptococcus pneumoniae sensibile ai macrolidi
116	streptococcus, altri
117	cocchi gram +, altri
118	neisseria meningiditis
119	moraxella (moraxella catarrhalis, moraxella spp)
120	listeria monocytogenes
121	neisseria gonorrhoeae
122	bacilli gram +, altri (bacillus cereus, bacillus spp, corynebacterium spp, lactobacillus, rhodococcus equi, nocardia spp, altro)
200	gram negativi
201	escherichia coli, sensibilità/resistenza non nota
202	escherichia coli sensibile ai beta-lattamici (tra cui cefalosporine di terza generazione)
203	escherichia coli resistente ai beta lattamici (tra cui cefalosporine di terza generazione)
204	escherichia coli resistente ai carbapenemici
205	enterobacter (di qualsiasi tipo)
206	klebsiella, sensibilità/resistenza non nota
207	klebsiella (qualsiasi tipo) sensibile ai beta-lattamici (tra cui cefalosporine di terza generazione)
208	klebsiella (qualsiasi tipo) resistente ai beta lattamici (tra cui cefalosporine di terza generazione)
209	klebsiella resistente ai carbapenemi
210	proteus o providencia (qualsiasi tipo)
211	salmonella (qualsiasi tipo)
212	serratia
213	citrobacter
214	pseudomonas aeruginosa, sensibilità/resistenza non nota
215	pseudomonas aeruginosa sensibile ai carbapenemi
216	pseudomonas aeruginosa sensibile ai beta lattamici (compresa terza generazione di cefalosporine)
217	pseudomonas aeruginosa resistente ai carbapenemi
218	pseudomonas aeruginosa resistente ai beta lattamici (tra cui cefalosporine di terza generazione)
219	pseudomonas, altri
220	acinetobacter, sensibilità/resistenza non nota
221	acinetobacter sensibile ai carbapenemi
222	acinetobacter resistente ai carbapenemi
223	stenotrophomonas maltophilia
224	campylobacter - helicobacter - brucella
225	haemophilus (influenzae o altro)
226	enterobatteri, altri (yersinia spp, shigella spp, altro)
227	eventuali batteri gram neg resistenti alla colistina
228	gram negativi, altri

300	anaerobi
301	clostridium (clostridium difficile, clostridium perfringes, clostridium spp, actinomyces, propionibacterium)
302	cocchi anaerobi (peptococcus, peptostreptococcus, veillonella)
303	bacteroidi (bacteroides fragilis, bacteroides melaninogenicus, capnocytophaga, fusobacterium spp,…)
304	anaerobi, altri
400	altri microrganismi
401	micobatteri (tubercolosi o altri)
402	clamidia
403	rickettsia
404	mycoplasma (mycoplasma pneumoniae o hominis, rochalimeae spp, bartonella spp)
405	legionella pneumoniae
500	funghi
501	candida albicans, sensibilità/resistenza non nota
502	candida albicans, sensibile ad azoli
503	candida albicans, resistente ad azoli
504	candida tropicalis, sensibilità/resistenza non nota
505	candida tropicalis, sensibile ad azoli
506	candida tropicalis, resistente ad azoli
507	candida glabrata, sensibilità/resistenza non nota
508	candida glabrata, sensibile ad azoli
509	candida glabrata, resistente ad azoli
510	candida krusei, sensibilità/resistenza non nota
511	candida krusei, sensibile ad azoli
512	candida krusei, resistente ad azoli
513	candida kefyr, sensibilità/resistenza non nota
514	candida kefyr, sensibile ad azoli
515	candida kefyr, resistente ad azoli
516	candida parapsilosis, sensibilità/resistenza non nota
517	candida parapsilosis, sensibile ad azoli
518	candida parapsilosis, resistente ad azoli
519	candida guilliermondii, sensibilità/resistenza non nota
520	candida guilliermondii, sensibile ad azoli
521	candida guilliermondii, resistente ad azoli
522	candida dubliniensis, sensibilità/resistenza non nota
523	candida dubliniensis, sensibile ad azoli
524	candida dubliniensis, resistente ad azoli
525	aspergillus
526	funghi, altri (cryptococcus neoformans, histoplasma spp,…)
600	virus
601	influenza A
602	influenza B
603	HSV I o II
604	CMV
605	altri
700	parassiti
701	plasmodium falciparum, pneumocistis carinii, toxoplasma gondii,…
801	flora mista

ANTIMICROBIAL LEGEND	
10	cefalosporine
11	cefazolina
12	cefuroxime
13	ceftazidime
14	ceftriaxone
15	cefepime/cefpirome
16	altra cefalosporina
20	penicilline
21	benzil penicillina
22	ampicillina
23	amoxy + clavulanato
24	pipera + tazo
25	oxa/cloxa/flucloxacillina
26	altra penicillina
30	carbapenemi
31	imipenem
32	meropenem
33	ertapenem
34	doripenem
35	altro carbapeneme
40	altri beta-lattami
41	temocillin
42	aztreonam
43	altri
50	aminoglicosidi
51	amikacina
52	tobramicina
53	gentamicina
54	altro aminoglicoside

60	chinoloni
61	ciprofloxacina
62	levofloxacina
63	altro chinolone
70	glicopeptidi
71	vancomicina
72	teicoplanina
73	altro glicopeptide
80	macrolidi
81	eritromicina
82	altro macrolide
90	altri antibiotici
91	metronidazolo
92	cotrimoxazolo
93	linezolid
94	daptomicina
95	tigeciclina
96	altri antibiotici
100	antifungini
101	fluconazolo
102	amfotericina B
103	amfotericina B formulazione lipidica
104	echinocandine
105	voriconazolo
106	altri antifungini
200	farmaci antivirali
201	HAART
202	oseltamivir
203	zanamivir
204	aciclovir
205	gangiclovir
206	amantadina
207	altri antivirali

[1] **Evidence of infection**: infections are considered 'definite', 'probable', or 'possible', according to the International Sepsis Forum Consensus Conference definitions.

[2] **Acquisition mode**: infections occurring at least 48h after hospital admission are considered 'hospital-acquired/health care-associated'; infections occurring at least 24h after ICU admission are considered 'ICU-acquired'; all other infections are considered 'community-acquired', according to the ECDC definitions.
