# Supplementary material for: Effectiveness of an Active and Continuous Surveillance Program for Intensive Care Units Infections Based on the EPIC III (Extended Prevalence of Infection in Intensive Care) Approach
Source: J Clin Med. 2022 Apr 28;11(9):2482. doi: 10.3390/jcm11092482 (PMC9101920; doi:10.3390/jcm11092482)
Supplement: Supplementary file 1 [file jcm-11-02482-s001.zip › SPRINT_supplemetary_Table S1A,B.pdf]

Table S1. (**A**). Characteristics of patients according to the ICU type. (**B**). Characteristics of infection according to the ICU type.

| (A) | | | | | | | | | | |
|---|---|---|---|---|---|---|---|---|---|---|
| | | **All patients (n=435)** | **ICU** | | | | | | | |
| | | | **General (n=60)** | **Cardiosurgical (n=76)** | **Traumatologic (n=104)** | **Post-operative (n=82)** | **Neurosurgical (n=39)** | **Pediatric (n=30)** | **Emergency department (n=26)** | **Gynecologic/ Obstetrical (n=18)** |
| Age, year, mean (SD) | | 58 (20.6) | 60 (10.8) | 63 (10.1) | 58 (18.8) | 64 (14.2) | 63 (16.6) | 4 (4.9) | 67 (12.2) | 57 (19.2) |
| Male, n (%) | | 261 (60.0) | 43 (71.7) | 50 (65.8) | 71 (68.3) | 51 (62.2) | 16 (41.0) | 16 (53.3) | 14 (53.8) | 0 (0) |
| Type of admission, n (%) | Medical | 120 (27.6) | 39 (65.0) | 8 (10.5) | 30 (28.8) | 8 (9.8) | 11 (28.2) | 15 (50.0) | 7 (26.9) | 2 (11.1) |
| | Surgical election | 158 (36.3) | 10 (16.7) | 56 (73.7) | 9 (8.7) | 54 (65.9) | 5 (12.8) | 10 (33.3) | 1 (3.8) | 13 (72.2) |
| | Surgical emergency | 105 (24.1) | 9 (15.0) | 12 (15.8) | 17 (16.3) | 20 (24.4) | 22 (56.4) | 4 (13.3) | 18 (69.2) | 3 (16.7) |
| | Trauma | 52 (12.0) | 2 (3.3) | 0 (0) | 48 (46.2) | 0 (0) | 1 (2.6) | 1 (3.3) | 0 (0) | 0 (0) |
| Reason for admission, n (%) | Respiratory | 57 (13.1) | 29 (48.3) | 4 (5.3) | 10 (9.6) | 2 (2.4) | 0 (0) | 11 (36.7) | 1 (3.8) | 0 (0) |
| | Cardiovascular | 55 (12.6) | 16 (26.7) | 15 (19.7) | 4 (3.8) | 9 (11.0) | 1 (2.6) | 1 (3.3) | 9 (34.6) | 0 (0) |
| | Neurological | 80 (18.4) | 8 (13.3) | 1 (1.3) | 16 (15.4) | 8 (9.8) | 37 (94.9) | 5 (16.7) | 5 (19.2) | 0 (0) |
| | Trauma | 57 (13.1) | 2 (3.3) | 0 (0) | 54 (51.9) | 0 (0) | 0 (0) | 0 (0) | 1 (3.8) | 0 (0) |
| | Surveillance | 154 (35.4) | 4 (6.7) | 55 (72.4) | 11 (10.6) | 56 (68.3) | 0 (0) | 13 (43.3) | 1 (3.8) | 14 (77.8) |
| | Other | 32 (7.4) | 1 (1.7) | 1 (1.3) | 9 (8.7) | 7 (8.5) | 1 (2.6) | 0 (0) | 9 (34.6) | 4 (22.2) |
| Comorbidities, yes, n (%) | | 274 (63.0) | 41 (68.3) | 62 (81.6) | 38 (36.5) | 74 (90.2) | 21 (53.8) | 7 (23.3) | 18 (69.2) | 13 (72.2) |
| SOFA, mean (SD) [a] | | 5 (4.1) | 6 (3.9) | 7 (4.6) | 4 (4.1) | 4 (3.0) | 6 (2.9) | 6 (0) | 9 (4.4) | 1 (1.2) |
| Invasive ventilation, n (%) | | 217 (49.9) | 39 (65.0) | 51 (67.1) | 47 (45.2) | 19 (23.2) | 22 (56.4) | 16 (53.3) | 23 (88.5) | 0 (0) |
| Non-invasive ventilation, n (%) | | 35 (8.0) | 5 (8.3) | 8 (10.5) | 3 (2.9) | 7 (8.5) | 1 (2.6) | 6 (20.0) | 0 (0) | 5 (27.8) |
| Tracheostomy, n (%) | | 114 (26.2) | 16 (26.7) | 16 (21.1) | 45 (43.3) | 9 (11.0) | 25 (64.1) | 1 (3.3) | 2 (7.7) | 0 (0) |
| Vasopressor use, n (%) | | 114 (26.2) | 15 (25.0) | 46 (60.5) | 15 (14.4) | 11 (13.4) | 10 (25.6) | 2 (6.7) | 15 (57.7) | 0 (0) |
| CVC, n (%) | | 372 (85.5) | 52 (86.7) | 73 (96.1) | 92 (88.5) | 71 (86.6) | 33 (84.6) | 17 (56.7) | 25 (96.2) | 9 (50.0) |
| Urinary catheter, n (%) | | 407 (93.6) | 54 (90.0) | 74 (97.4) | 96 (92.3) | 76 (92.7) | 37 (94.9) | 26 (86.7) | 26 (100.0) | 18 (100.0) |
| Renal replacement therapy, n (%) | | 39 (9.0) | 13 (21.7) | 13 (17.1) | 4 (3.8) | 5 (6.1) | 1 (2.6) | 2 (6.7) | 1 (3.8) | 0 (0) |
| ECMO, n (%) | | 8 (1.8) | 5 (8.3) | 3 (3.9) | 0 (0) | 0 (0) | 0 (0) | 0 (0) | 0 (0) | 0 (0) |
| Septic shock, n (%) | | 69 (15.9) | 14 (23.3) | 23 (30.3) | 9 (8.7) | 4 (4.9) | 7 (17.9) | 0 (0) | 12 (46.2) | 0 (0) |
| Hyperlactacidemia, n (%) | | 77 (17.7) | 5 (8.3) | 32 (42.1) | 4 (3.8) | 18 (22.0) | 0 (0) | 2 (6.7) | 13 (50.0) | 3 (16.7) |
| ICU length of stay, days, median (IQR) [a] | | 14 (4-36) | 21 (4-57) | 7 (2-31) | 26 (10-32) | 4 (1-9) | 26 (10-39) | 8 (2-15) | 7 (0-21) | 4 (2-5) |
| Hospital length of stay, days, median (IQR) [a] | | 29 (15-54) | 53 (28-72) | 27 (15-49) | 58 (36-68) | 21 (11-43) | 50 (18-57) | 36 (15-62) | 34 (11-44) | 11 (7-18) |
| Mortality at 60 days, n (%)[a] | | 52 (12.9) | 10 (18.2) | 11 (15.7) | 11 (11.1) | 6 (7.5) | 1 (2.9) | 2 (7.4) | 10 (47.6) | 1 (5.9) |
| (B) | | | | | | | | | | |
| | | **All patients** | **ICU** | | | | | | | |

| | | (n=435) | **General (n=60)** | **Cardiosurgical (n=76)** | **Traumatologic (n=104)** | **Post-operative (n=82)** | **Neurosurgical (n=39)** | **Pediatric (n=30)** | **Emergency department (n=26)** | **Gynecologic/ Obstetrical (n=18)** |
|---|---|---|---|---|---|---|---|---|---|---|
| Infection, n (%) | | 184 (42.3) | 47 (78.3) | 31 (40.8) | 42 (40.4) | 15 (18.3) | 21 (53.8) | 12 (40.0) | 15 (57.7) | 1 (5.6) |
| Antibiotic prophylaxis, n (%) | | 149 (34.3) | 6 (10.0) | 38 (50.0) | 9 (8.7) | 58 (70.7) | 4 (10.3) | 10 (33.3) | 8 (30.8) | 16 (88.9) |
| Antibiotic therapy, n (%) | | 162 (37.2) | 39 (65.0) | 30 (39.5) | 34 (32.7) | 15 (18.3) | 16 (41.0) | 12 (40.0) | 15 (57.7) | 1 (5.6) |
| Positive Isolates, n (%) | Gram-positive | 34 (7.8) | 7 (11.7) | 5 (6.6) | 5 (4.8) | 5 (6.1) | 8 (20.5) | 3 (10.0) | 1 (3.8) | 0 (0) |
| | Gram-positive MS | 19 (4.4) | 2 (3.3) | 3 (3.9) | 3 (2.9) | 2 (2.4) | 6 (15.4) | 3 (10.0) | 0 (0) | 0 (0) |
| | Gram-positive MDR | 17 (3.9) | 5 (8.3) | 2 (2.6) | 2 (1.9) | 3 (3.7) | 4 (10.3) | 0 (0) | 1 (3.8) | 0 (0) |
| | Gram-negative | 98 (22.5) | 23 (38.3) | 13 (17.1) | 29 (27.9) | 10 (12.2) | 12 (30.8) | 7 (23.3) | 4 (15.4) | 0 (0) |
| | Gram-negative MS | 69 (15.9) | 15 (25.0) | 8 (10.5) | 19 (18.3) | 6 (7.3) | 12 (30.8) | 7 (23.3) | 2 (7.7) | 0 (0) |
| | Gram-negative MDR | 47 (10.8) | 14 (23.3) | 8 (10.5) | 16 (15.4) | 6 (7.3) | 1 (2.6) | 0 (0) | 2 (7.7) | 0 (0) |
| | All MDR bacteria | 59 (13.6) | 16 (26.7) | 10 (13.2) | 18 (17.3) | 7 (8.5) | 5 (12.8) | 0 (0) | 3 (11.5) | 0 (0) |
| | Fungi | 19 (4.4) | 11 (18.3) | 1 (1.3) | 3 (2.9) | 2 (2.4) | 1 (2.6) | 0 (0) | 1 (3.8) | 0 (0) |
| | Viruses | 8 (1.8) | 4 (6.7) | 1 (1.3) | 1 (1.0) | 1 (1.2) | 0 (0) | 1 (3.3) | 0 (0) | 0 (0) |
| | *Klebsiella* | 40 (9.2) | 12 (20.0) | 8 (10.5) | 10 (9.6) | 5 (6.1) | 1 (2.6) | 2 (6.7) | 2 (7.7) | 0 (0) |
| | *Pseudomonas* | 30 (6.9) | 12 (20.0) | 2 (2.6) | 10 (9.6) | 2 (2.4) | 2 (5.1) | 1 (3.3) | 1 (3.8) | 0 (0) |
| | *Acinetobacter* | 16 (3.7) | 2 (3.3) | 2 (2.6) | 8 (7.7) | 1 (1.2) | 3 (7.7) | 0 (0) | 0 (0) | 0 (0) |
| | Bacteria resistant to Carbapenems | 36 (8.3) | 11 (18.3) | 6 (7.9) | 13 (12.5) | 5 (6.1) | 0 (0) | 0 (0) | 1 (3.8) | 0 (0) |
| Site of infection, n (%) | Respiratory system | 114 (26.2) | 30 (50.0) | 21 (27.6) | 20 (19.2) | 12 (14.6) | 20 (51.3) | 6 (20.0) | 5 (19.2) | 0 (0) |
| | Abdomen | 21 (4.8) | 8 (13.3) | 0 (0) | 2 (1.9) | 2 (2.4) | 0 (0) | 1 (3.3) | 8 (30.8) | 0 (0) |
| | Circulation | 69 (15.9) | 20 (33.3) | 17 (22.4) | 17 (16.3) | 5 (6.1) | 1 (2.6) | 3 (10.0) | 6 (23.1) | 0 (0) |
| | Kidney/genitourinary | 17 (3.9) | 6 (10.0) | 0 (0) | 4 (3.8) | 1 (1.2) | 4 (10.3) | 1 (3.3) | 0 (0) | 1 (5.6) |
| | Others | 26 (6.0) | 6 (10.0) | 1 (1.3) | 14 (13.5) | 2 (2.4) | 0 (0) | 3 (10.0) | 0 (0) | 0 (0) |
| Acquisition mode, n (%) | Community-acquired | 36 (8.3) | 16 (26.7) | 3 (3.9) | 4 (3.8) | 2 (2.4) | 0 (0) | 6 (20.0) | 5 (19.2) | 0 (0) |
| | Hospital-acquired/Health Care-associated | 41 (9.4) | 6 (10.0) | 5 (6.6) | 9 (8.7) | 5 (6.1) | 6 (15.4) | 3 (10.0) | 6 (23.1) | 1 (5.6) |
| | ICU-acquired | 117 (26.9) | 25 (41.7) | 23 (30.3) | 34 (32.7) | 11 (13.4) | 16 (41.0) | 4 (13.3) | 4 (15.4) | 0 (0) |
| Evidence of Infection, n (%) | Certain | 108 (24.8) | 31 (51.7) | 15 (19.7) | 35 (33.7) | 9 (11.0) | 6 (15.4) | 8 (26.7) | 4 (15.4) | 0 (0) |
| | Probable | 44 (10.1) | 11 (18.3) | 11 (14.5) | 5 (4.8) | 5 (6.1) | 4 (10.3) | 1 (3.3) | 7 (26.9) | 0 (0) |
| | Feasible | 52 (12.0) | 8 (13.3) | 8 (10.5) | 9 (8.7) | 4 (4.9) | 14 (35.9) | 3 (10.0) | 5 (19.2) | 1 (5.6) |

CVC: central venous catheter; ECMO: Extracorporeal membrane oxygenation; ICU: Intensive care unit; IQR: interquartile range; SD: standard deviation; SOFA: Sequential Organ Failure Assessment score; MDR: multi-drug resistant; MS: multi-sensitive. * Significant at 5% level; [a] Total patients is not 435 because of missing values. Percentages are calculated considering missing values. Percentages can exceed 100% because patients could have more than one infection.
